# Sunburns among beachgoers in the northern coast of Peru: frequency and factors associated

**DOI:** 10.7717/peerj.11473

**Published:** 2021-06-09

**Authors:** Eliana L. Fernandez-Quiroz, Lizeth Gonzales-Chachapoyas, Ana L. Alcantara-Diaz, Binz Bulnes-Villalta, Zulmy Ayala-Porras, Carlos J. Toro-Huamanchumo

**Affiliations:** 1 Universidad Católica Santo Toribio de Mogrovejo, Chiclayo, Peru; 2 Asociación Científica de Estudiantes de Medicina de la Universidad Católica Santo Toribio de Mogrovejo - ASOCIEM USAT, Chiclayo, Peru; 3 Sociedad Científica de Estudiantes de Medicina Veritas, Chiclayo, Peru; 4 Universidad de San Martín de Porres, Facultad de Medicina Humana, Chiclayo, Peru; 5 Unidad para la Generación y Síntesis de Evidencias en Salud, Universidad San Ignacio de Loyola, Lima, Peru

**Keywords:** Sunburn, Ultraviolet rays, Sunscreen, Skin, Peru

## Abstract

**Background:**

Overexposure to ultraviolet (UV) radiation has increased skin cancer incidence and the risk of sunburns, especially during the summer months.

**Objective:**

Identify the frequency and factors associated with sunburns in a sample of beachgoers in the northern coast of Peru.

**Methods:**

We conducted a secondary data analysis of a previous study that assessed the awareness, behavior and attitudes concerning sun exposure among beachgoers. We included adults between 18 and 59 years who went to a beach in northern Peru during summer (March 2018). Three generalized linear models of the Poisson family were constructed to evaluate the factors associated with having had at least one sunburn last summer. All regression models reported the adjusted prevalence ratio (aPR) with their respective 95% confidence interval (95% CI).

**Results:**

Of a total of 402 participants, 225 (56.0%) had one to five sunburns and 25 (6.2%) had six or more. Beachgoers who were 1–15 days (aPR: 1.16, 95% CI [1.05–1.27]) or more than 15 days (aPR: 1.22, 95% CI [1.09–1.36]) exposed to the sun on the beach had a higher frequency of at least one sunburn. The non-regular wearing of a hat or cap also increased the frequency of sunburns (aPR: 1.06, 95% CI [1.01–1.12]). In contrast, those who had Skin Phototype III (aPR: 0.94, 95% CI [0.88–0.99]) or IV (aPR: 0.69, 95% CI [0.63–0.75]) had a lower frequency of sunburns.

**Conclusion:**

Three out of five beachgoers had one or more sunburns in the last summer. The factors associated with a higher frequency were the time of sun exposure at the beach and the non-regular use of a hat or cap. Type III–IV skin phototypes were associated with a lower sunburn frequency.

## Introduction

Skin cancer incidence has increased in the last decades (Apalla et al., 2017). In 2018, more than a million new non-melanoma skin cancer cases were reported worldwide (Bray et al., 2018), and for melanoma, there is an estimate of 132,000 new cases per year (World Health Organization, 2017). Additionally, according to current evidence, the economic burden per patient who receives skin cancer treatment is considerably high, making it a significant public health problem (Guy et al., 2015; Gordon et al., 2016; Doran et al., 2015).

Peru is one of the countries with the highest ultraviolet (UV) radiation due to its proximity to the equator (Liley & McKenzie, 2006). The National Meteorology and Hydrology Service in Peru (SENAMHI, in Spanish) has confirmed that the UV radiation index has reached levels as high as 19, on a scale of 0–20, mainly in coastal regions (Senamhi, 2017). In addition, skin cancer has risen from fourth place (period 2006–2011) (Ministerio de Salud, 2013) to third place in the list of the most frequent types of cancer independent of sex (in 2017) (Ministerio de Salud, 2018).

Current evidence has shown the relationship between overexposure to UV radiation and skin cancer (Holman et al., 2018; Bruce, Theeke & Mallow, 2017). This risk factor is usually more frequent in people who go to the beaches during the summer season (O’Riordan et al., 2008), representing a high-risk population. Previous studies have shown that beachgoers have less adherence to the use of sun protection measures than other populations, additionally to their prolonged exposure to UV radiation in summer months (Heerfordt et al., 2017; de Troya-Martín et al., 2014; Cercato et al., 2015; Robinson et al., 2016). These inappropriate attitudes and practices may act as risk factors for sunburns and later development of skin cancer (Marion et al., 2018; Diffey, 2018; Holman et al., 2019).

Previous studies conducted in adult populations have reported a frequent lifetime history of at least one sunburn (Holman et al., 2018; Arutyunyan et al., 2017; Pinault & Fioletov, 2017), with beachgoers as the highest risk group (Toro-Huamanchumo et al., 2019; Troya-Martín et al., 2018). Sun sensitivity, a younger age, having a high perceived vulnerability to skin cancer, and having had a full-body skin examination by a physician have been reported as factors associated with sunburns among US adults (Holman et al., 2018; Arutyunyan et al., 2017). Similarly, a younger age, male sex, secondary or university education, skin phototypes I–III, midday sun exposure, and lousy sun protection habits have been reported as predictors of sunburns among Spanish beachgoers (Troya-Martín et al., 2018). However, we have not found local studies that assess the association between modifiable and non-modifiable risk factors with sunburns, especially among this population of interest.

This study aimed to determine the frequency and factors associated with sunburns in a sample of beachgoers in the northern coast of Peru.

## Methods

### Study design

This is a secondary data analysis of a previous study that assessed the awareness, behavior and attitudes concerning sun exposure among beachgoers in the northern coast of Peru (Toro-Huamanchumo et al., 2019).

### Study population and procedures

We selected from the database the adults surveyed during their visit to Pimentel, which has one of the most crowded beaches in Lambayeque. This region has a semi-warm and tropical-dry climate, with temperatures peaks of 30 °C since 2014 (Senamhi, 2017; Instituto Nacional de Defensa Civil, 2003).

For the primary study, a total of 410 participants ≥18 years old were enrolled using a convenience sampling strategy. The research team applied the “Beach Questionnaire” during the summer season (March 2018), between 08:00 and 16:00 hours. More information about the study population, context and procedures has been previously described in this journal (Toro-Huamanchumo et al., 2019). We decided to include only adults between 18–59 years old (*n* = 402) for the present study. We did not have missing values.

### Outcome

The outcome variable “sunburn” was defined as an episode of painful reddening of the skin after sun exposure in the last summer (Troya-Martín et al., 2018).

### Independent variables

The following independent variables were included in the analysis: (1) Sociodemographic variables: age (young adult: 18–29 years, and adult: 30–59 years), sex (male and female), nationality, level of education (None/school and higher education), marital status (single/widowed/divorced/separated and married/cohabiting); (2) Skin phototype from the Fitzpatrick model and classified into four categories (I–IV) according to the erythema and tanning response after one hour of sun exposure in the summer (Troya-Martín et al., 2018; Sánchez & Nova, 2008); (3) Sun exposure habits in the last two summers were assessed as: days spent sunbathing on the beach (none, 1–15 days, and >15 days), and hours per day exposed to the sun on the beach (<30 min, 30 min to 1 h, and >1 h); (4) Sun protection practices in the beach (use of an umbrella, wear a hat or cap, wear long-sleeved clothes, avoid sun exposure in hours between 12:00 and 16:00), and use of sunscreen with a sun protection factor ≥15. These sun protection practices were dichotomized into regular (always or usually) and non-regular (sometimes, rarely, or never). We did not include the variable “use of sunglasses” (as in the primary study) since we consider there is no plausible relationship between this variable and sunburns.

### Statistical analysis

According to the history of sunburns in the last summer (none and ≥1 sunburn ). For categorical variables, we used Chi-square and Fisher tests. To assess the factors associated with having had at least one sunburn in the last summer (dichotomous variable), we constructed three generalized linear models (GLM) of the Poisson family with a log link function and robust standard errors. We decided to use the Poisson family instead of logistic regression to avoid overestimating the calculated associations, especially because the prevalence of sunburns (outcome) was >10% (Barros & Hirakata, 2003; Tamhane et al., 2016). Model 1 included sociodemographic characteristics (age, sex, marital status, and level of education). Model 2 included Model 1 + sun exposure habits and practices. Finally, model 3 included model 2 + the skin phototype variable. We opted for this selection process in order to achieve better control of confounding factors. In addition, we compared the final model with de Model 2 using the log-likelihood ratio test. Since we obtained a *p* < 0.05, we considered that the block of variables included in Model 3 was relevant to explaining the outcome.

All regression models reported the adjusted prevalence ratio (aPR) with their respective 95% confidence intervals (95% CI). The statistical analysis was carried out using the statistical package Stata v15.0 (StataCorp, College Station, TX, USA).

### Ethics

The Institutional Review Board of the Hospital San Bartolomé (RCEI-40) in Lima, Peru, approved the primary study. Participation in the survey was voluntary, and all the participants provided their oral consent.

## Results

### Characteristics of the study population and history of sunburns

We analyzed a total of 402 beachgoers of both sexes. A total of 232 (57.7%) were female, 152 (37.8%) had no sunburn, 225 (56.0%) had one to five sunburns and 25 (6.2%) had six or more. Table 1 shows the study population’s characteristics according to the number of sunburns in the last summer.

**Table 1:** Sociodemographic characteristics, skin phototype, habits, and sun protection practices, according to sunburn history (*n* = 402).

Variables	Sunburn history		*p*
	None (*n* = 152)	≥1 (*n* = 250)	
Age			0.543**
Young adult	81 (36.5)	141 (63.5)	
Adult	71 (39.4)	109 (60.6)	
Sex			0.528**
Male	62 (36.1)	110 (63.9)	
Female	90 (39.1)	140 (60.9)	
Marital status			0.635**
Single/Widowed/Divorced/separated	90 (38.8)	142 (61.2)	
Married or cohabiting	62 (36.5)	108 (63.5)	
Nationality			0.403^†^
Peru	148 (37.6)	246 (62.4)	
Argentina	2 (100.0)	0 (0.0)	
Colombia	1 (33.3)	2 (66.7)	
Ecuador	1 (50.0)	1 (50.0)	
Mexico	0 (0.0)	1 (100.0)	
Level of education			0.606**
None or school	58 (39.5)	89 (60.5)	
Higher education	94 (36.9)	161 (63.1)	
Skin phototype			<0.001**
I	11 (18.3)	49 (81.7)	
II	22 (27.8)	57 (72.2)	
III	47 (28.8)	116 (71.2)	
IV	72 (72.0)	28 (28.0)	
*Sun exposure habits in the last two summers:*			
Days spent sunbathing on the beach			0.001**
None	32 (59.3)	22 (40.7)	
1 - 15 days	101 (35.8)	181 (64.2)	
More than 15 days	19 (28.8)	47 (71.2)	
Hours per day exposed to the sun on the beach			0.025**
Less than 30 minutes	35 (52.2)	32 (47.8)	
30 minutes to one hour	35 (37.2)	59 (62.8)	
More than one hour	82 (34.0)	159 (66.0)	
*Sun protection practice in the beach:*			
Non-regular using of a beach umbrella	88 (39.1)	137 (60.9)	0.544**
Non-regular wearing of a hat or cap	76 (35.7)	137 (64.3)	0.350**
Non-regular wearing long-sleeved clothing	120 (37.0)	204 (63.0)	0.514**
Non-regular avoiding of sun exposure during the midday	75 (32.1)	159 (68.0)	0.005**
Non-regular using of sunscreen with a SPF ≥ 15	50 (34.2)	96 (65.8)	0.266**

**Notes:**

** Chi2 test.

† Fisher exact test.

SPF: Sun protection factor.

### Factors associated with sunburns

Table 2 shows the Poisson regression models’ results for assessing the factors associated with having had at least one sunburn in the last summer. In Model 1, no associated factors were identified. In model 2, some associated factors were added, such as not avoiding sun exposure during midday (*p* = 0.019) and having sunbathed on the beach from one to 15 days (*p* = 0.011) or more than 15 days (*p* = 0.004) in the last two summers. Finally, Model 3 showed that the frequency of having had at least one sunburn in the last summer was significantly higher among those with a non-regular wearing of hat or cap (*p* = 0.040), and those who spent one to 15 days (*p* = 0.003) or more than 15 days (*p* < 0.001) sunbathing on the beach. On the other hand, having at least one sunburn was less frequent with skin phototype III (*p* = 0.038) or IV (*p* < 0.001).

**Table 2:** Factors associated with sunburns (*n* = 402).

Variables	Model 1			Model 2			Model 3		
	aPR	95% CI	*p*	aPR	95% CI	*p*	aPR	95% CI	*p*
Age (adults)	0.96	[0.90–1.03]	0.300	0.98	[0.91–1.05]	0.493	0.98	[0.93–1.05]	0.614
Sex (females)	0.98	[0.92–1.04]	0.527	1.01	[0.95–1.07]	0.895	0.99	[0.94–1.05]	0.786
Marital status									
Single	Ref.			Ref.			Ref.		
Married or cohabiting	1.04	[0.97–1.11]	0.291	1.03	[0.96–1.10]	0.380	0.99	[0.93–1.05]	0.662
Leve of education									
None or school	Ref.			Ref.			Ref.		
Higher education	1.01	[0.95–1.08]	0.705	1.03	[0.97–1.09]	0.403	0.99	[0.93–1.04]	0.643
Days spent sunbathing on the beach in the last two summers									
None				Ref.			Ref.		
1–15 days				1.14	[1.03–1.26]	0.011	**1.16**	**[1.05–1.27]**	**0.003**
More than 15 days				1.19	[1.06–1.35]	0.004	**1.22**	**[1.09–1.36]**	**<0.001**
Hours spent sunbathing on the beach in the last two summers									
Less than 30 minutes				Ref.			Ref.		
30 minutes to one hour				1.06	[0.96–1.17]	0.245	1.08	[0.98–1.19]	0.122
More than one hour				1.05	[0.96–1.15]	0.313	1.06	[0.97–1.16]	0.200
Non-regular using of a beach umbrella				0.98	[0.92–1.04]	0.411	0.98	[0.93–1.04]	0.509
Non-regular wearing of a hat or cap				1.02	[0.96–1.08]	0.551	**1.06**	**[1.01–1.12]**	**0.040**
Non-regular wearing long-sleeved clothing				1.01	[0.93–1.09]	0.856	1.02	[0.95–1.10]	0.609
Non-regular avoiding of sun exposure during midday				1.07	[1.01–1.14]	0.019	1.04	[0.99–1.10]	0.146
Non-regular using of sunscreen with a SPF ≥ 15				1.04	[0.98–1.11]	0.203	1.05	[1.00–1.11]	0.072
Skin phototype									
I							Ref.		
II							0.95	[0.89–1.02]	0.175
III							**0.94**	**[0.88–0.99]**	**0.038**
IV							**0.69**	**[0.63–0.75]**	**<0.001**

**Note:**

aPR, Adjusted prevalence ratio; SPF, Sun protection factor; 95% CI, 95% confidence intervals.

*p*-values ≤0.05 are in bold

## Discussion

### Main results

In our study sample, we found different factors associated with sunburns in people who went to a beach in northern Peru. Having skin phototype III or IV was associated with a lower frequency of sunburn. However, going to the beach for 1 to 15 days, more than 15 days, and not wearing a hat or cap regularly were associated with a higher frequency.

### Sunburns frequency

More than a half (62.2%) of the beachgoers reported having had at least one sunburn in the last two summers in our study sample. A previous study in beachgoers and skaters from Costa del Sol, Spain, found a lower sunburn frequency (46.9% and 56.8%, respectively) (de Troya-Martín et al., 2014; Toro-Huamanchumo et al., 2019). However, another study conducted in a sample of beach handball players from Spain found a higher sunburn frequency (76.9% had at least one sunburn in the last year) (De Castro-Maqueda et al., 2019). These sunburn events could be related to prolonged sun exposure among the populations mentioned, in addition to insufficient sun protection measures (DeFlorio-Barker et al., 2020).

### Factors associated with sunburns

Regarding the use of hats or caps, in our study, the non-regular use of these accessories was associated with a higher prevalence of sunburn. This result is explained by a previous study by Backes C et al (2018), in which the effectiveness of using hats for sun protection was assessed. They found that not wearing this accessory and inadequate face protection in the midday hours of summer were associated with receiving higher doses of solar ultraviolet radiation (UVR) (Backes et al., 2018).

Regarding the frequency of sunbathing on the beach in the last two summers, bathers who sunbathed at least one day had a higher frequency of sunburns. This frequency was even higher among those who sunbathed for more than 15 days. These are expected results since one person is more vulnerable to sunburns if there are no adequate sun protection measures and more prolonged sun exposure (Marion et al., 2018; Troya-Martín et al., 2018; Makin et al., 2013).

Beachgoers with a skin phototype type III–IV showed a lower frequency of sunburns in the last two summers. An explanation for this result is based on the Fitzpatrick’s study which showed that the skin phototype IV rarely suffers sunburns and develops a tan more easily. In addition, although adults with skin phototype III may also have some risk of suffering from sunburns, its risk is lower than skin phototypes type I and II (Coelho et al., 2009; Castanedo-Cazares et al., 2018).

### Public health relevance

Like previous studies (Holman et al., 2018; Troya-Martín et al., 2018; Sordo & Gutiérrez, 2013), our analysis highlights the presence of several problems, such as inadequate knowledge about sun protection, overexposure to the sun and a high prevalence of sunburns. Health policies that encourage sunscreen use and public health campaigns aimed to increase awareness about the health risks of UV exposure are urgently needed, emphasizing populations at risk (Strickland & Fritschi, 2014), such as beachgoers and tourists.

In Peru, every first Sunday of February, we celebrate “El día del lunar”, a national awareness day where medical campaigns are carried out along with informative talks. These activities’ main objective is to raise awareness about the health risks of prolonged sun exposure (Sordo & Gutiérrez, 2013) and skin cancer (Holman et al., 2018; Erdmann et al., 2013).

Peru must adopt evidence-based strategies. For example, in Australia, some cross-sectional studies assessed the impact of sun protection campaigns. These campaigns are delivered through the media during the summer, with favorable results reported (Smith et al., 2002; Koch et al., 2017). Likewise, these studies recognize that awareness campaigns contribute to the short-term increase of some sun protection behaviors. Similarly, New Zealand has middle school programs that include teaching preventing practices to avoid overexposure to the sun and developing appropriate attitudes and habits at an early age (McNoe & Reeder, 2019). In Latin America, there are incomplete and heterogeneous reports of the preventive measures adopted by the countries (Vries et al., 2016). Therefore, it is necessary to carry out studies that prospectively evaluate the impact of campaigns or other activities in reducing skin cancer incidence.

## Limitations

The present study has some limitations. First, the possibility of social desirability bias. The beachgoers could have responded according to the sun protection measures presented in the media or their previous knowledge, reporting sun protection habits that they do not practice. Second, we did not assess the effect of some variables that could potentially influence our study results, such as current or past illnesses and a family history of skin cancer. Third, our study does not represent the Pimentel district’s demography because many beachgoers come to vacation from other cities, regions, and countries during the summer. The study also does not necessarily represent the expected frequencies to be found in subsequent studies since population and climatic conditions may vary. Fourth, due to the cross-sectional design of the study, we cannot infer causality between the variables of interest.

Despite these limitations, this study is one of the first in the local context to assess factors associated with sunburns. There are previous studies that aimed to describe the knowledge, habits, and attitudes about photoprotection. However, they were conducted in different populations, such as outpatients, farmers, and company workers (Thomas-Gavelan et al., 2011; Cueva-Puelles et al., 2019; Mejía et al., 2018). Only two studies by Ramos W et al. assessed the knowledge, attitudes, and practices of sun protection in a sample of beachgoers and parents who went to beaches in Lima, Peru (Ramos et al., 2013; Ramos et al., 2017). Additionally, although our study has an exploratory approach, it allowed the construction of different models adjusting for blocks of variables, which would increase the robustness of our findings.

## Conclusion

Three out of five beachgoers had experience one or more sunburns in the last summer. The factors associated with a higher frequency were the time of sun exposure and the non-regular use of a hat or cap at the beach. Skin phototypes III–IV were associated with a lower frequency of sunburn.

## Additional Information and Declarations

### Data Availability

The following information was supplied regarding data availability: The database is available at figshare: Toro-Huamanchumo, Carlos (2018): Dataset.eng - Sun exposure. figshare. Dataset. DOI 10.6084/m9.figshare.6828725.v1.
